# Treatment of spontaneous osteonecrosis of the knee by daily teriparatide: A report of 3 cases

**DOI:** 10.1097/MD.0000000000018989

**Published:** 2020-01-31

**Authors:** Akira Horikawa, Naohisa Miyakoshi, Michio Hongo, Yuji Kasukawa, Yoichi Shimada, Hiroyuki Kodama, Akihisa Sano

**Affiliations:** a Shizuoka Tokusyukai Hospital, Shizuoka; b Department of Orthopedic Surgery, Akita University Graduate School of Medicine, Akita; c South Akita Orthopedic Clinic, Katagami, Japan.

**Keywords:** daily teriparatide, MRI, osteoporosis, SONK, VAS

## Abstract

**Rationale:**

Although the treatment of femoral head necrosis has already been established with the adoption of daily teriparatide, a clear consensus on the treatment of spontaneous osteonecrosis of the knee (SONK) has yet to be reached. Therefore, we focused on the treatment of SONK with daily teriparatide administration (20 μg, subcutaneous) and confirmed its effects to determine whether it is a valid option.

**Patients’ concerns:**

Three osteoporotic patients who were diagnosed with SONK complained of knee pain.

**Diagnosis:**

SONK was diagnosed on magnetic resonance imaging in all cases.

**Interventions:**

All patients took daily teriparatide as a treatment for SONK.

**Outcomes:**

There was a significant and dramatic reduction in the visual analog scale score 1 month after treatment. After 6 months of treatment, the sizes of the affected SONK lesions were smaller than in the initial phase, and plain X-rays showed no further signs of progression.

**Lessons:**

Daily teriparatide might be an effective treatment for SONK.

## 1 Introduction

There are many theories about the etiology of spontaneous osteonecrosis of the knee (SONK), but its treatment has not yet been established, although some effective treatments for osteoporosis have been reported. Treatment can be either operative or nonoperative. The operative approaches range from arthroscopy and osteotomy to knee arthroplasty, such as unicompartmental or total knee arthroplasty.^[1–5]^ Nonoperative management has recently reported the reduction of weight-bearing and the use of anti-inflammatory medication.^[6,7]^ Currently, many orthopedic surgeons prescribe bisphosphonates because they have been shown to be effective in treating localized bone metabolism disorders.^[8]^

In addition, there are some reports of the treatment of osteonecrosis of the femoral head with teriparatide, which promotes bone formation and prevents the collapse of subchondral bone.^[9]^ this report provides a new study on the efficacy of teriparatide for the treatment of SONK through the use of 3 patients.

## 2 Materials and methods

Three patients (2 women, 1 man) with SONK diagnosed on magnetic resonance imaging (MRI) were recruited from 2017 to 2018. All suffering from knee pain without severe osteoarthritic lesions on plain X-rays. Their health status, age, body mass index, bone mineral density (BMD), and bone metabolism were assessed. Ethical approval was waived because all of the patients had osteoporosis, and they sought daily teriparatide which was administrated by subcutaneous injection (20 μg every day) as the primary treatment for osteoporosis. Furthermore, our institution does not require ethical approval for studies reporting individual cases or case series. The patient(s) or a legally authorized representative provided written, informed consent for patient information and images to be published.

### 2.1 BMD

A DXA bone scan was performed to evaluate forearm BMD (DTX-200; Datex DSM, Courtaboeuf Cedex, France).

### 2.2 Radiology

Plain X-rays were performed for all patients, and osteoarthritis was classified by the Kellgren–Lawrence stage (KL stage), and the femoro-tibial angle (FTA) was measured in the standing position.

### 2.3 MRI

The affected lesions were measured as low and high-intensity areas on T2-weighted images, and the data were analyzed in coronal and sagittal views, as previously described.^[10]^

### 2.4 Bone turnover markers and serum calcium (Ca)

Serum tartrate-resistant acid phosphatase 5b (TRACP-5b, mU/dL), type 1 procollagen N-terminal propeptide (P1NP, μg/L), and serum Ca (mg/l) levels were measured at baseline, 6 months, and 12 months. The least significant change of each bone turnover marker was 14.1% and 23.5%, respectively.

### 2.5 Visual analog scale (VAS)

Patients’ symptoms were assessed using a VAS for pain at the initial phase and 1 month after daily teriparatide was started. The VAS ranged from zero to ten, with the patient reporting zero for minimal pain, and 10 as the worst possible pain.

## 3 Case reports

### 3.1 Case 1

**Figure 1:**
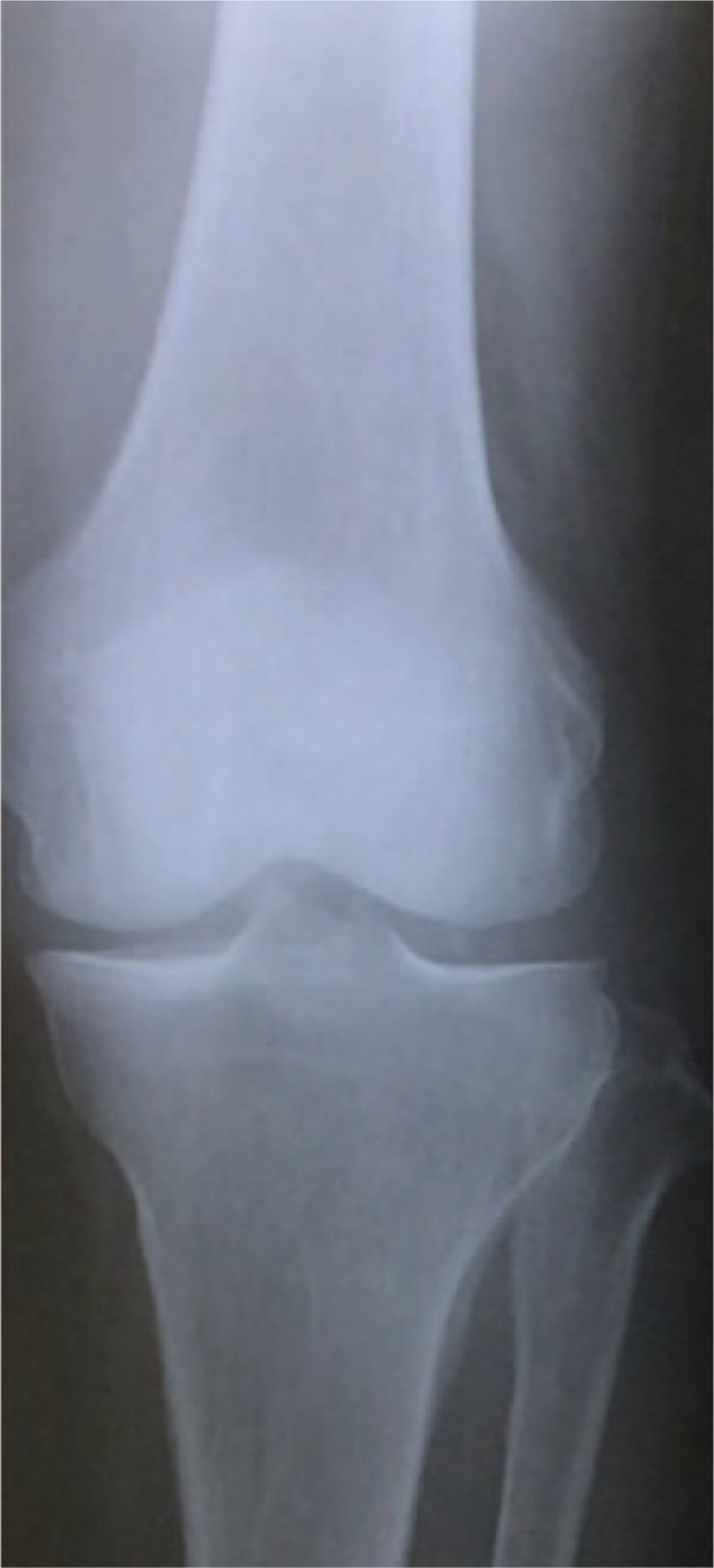
Case 1 in the initial phase. Plain X-ray shows no osteoarthritic lesion.

**Figure 2:**
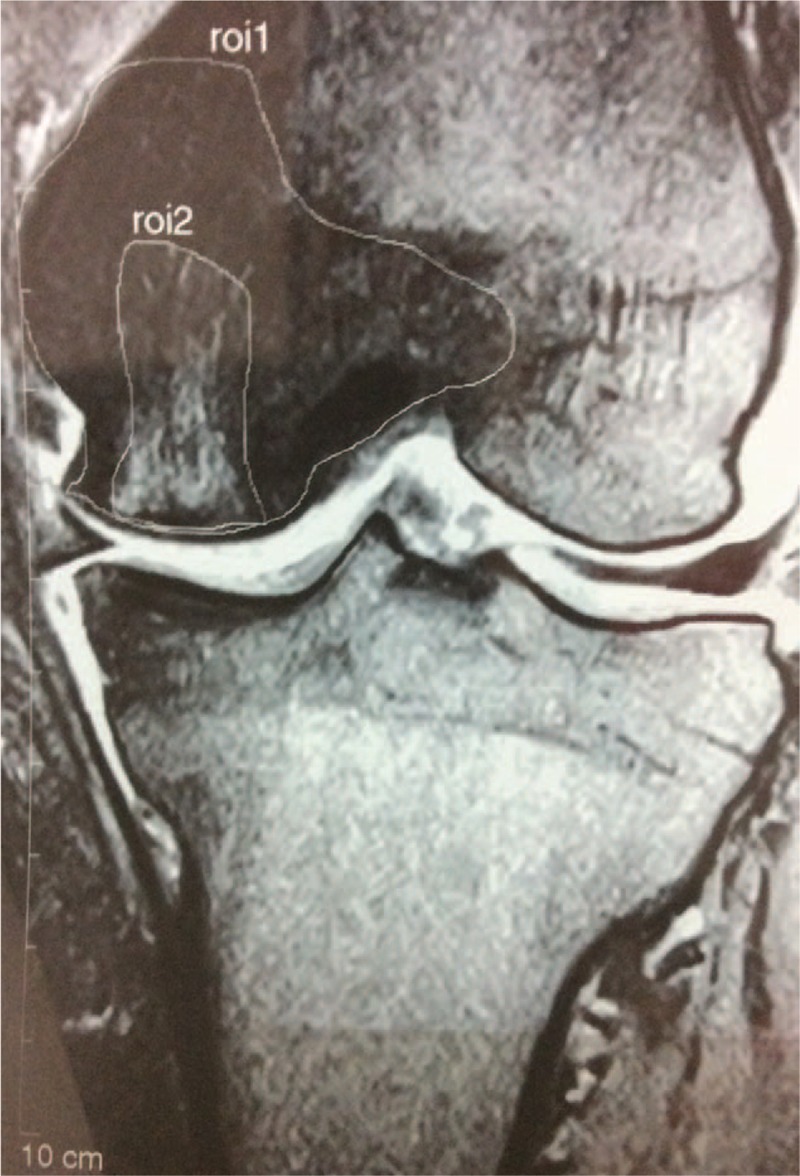
Case 1 in the initial phase. MRI shows low and high-intensity areas that identify the SONK lesion. MRI = magnetic resonance imaging, SONK = spontaneous osteonecrosis of the knee.

**Figure 3:**
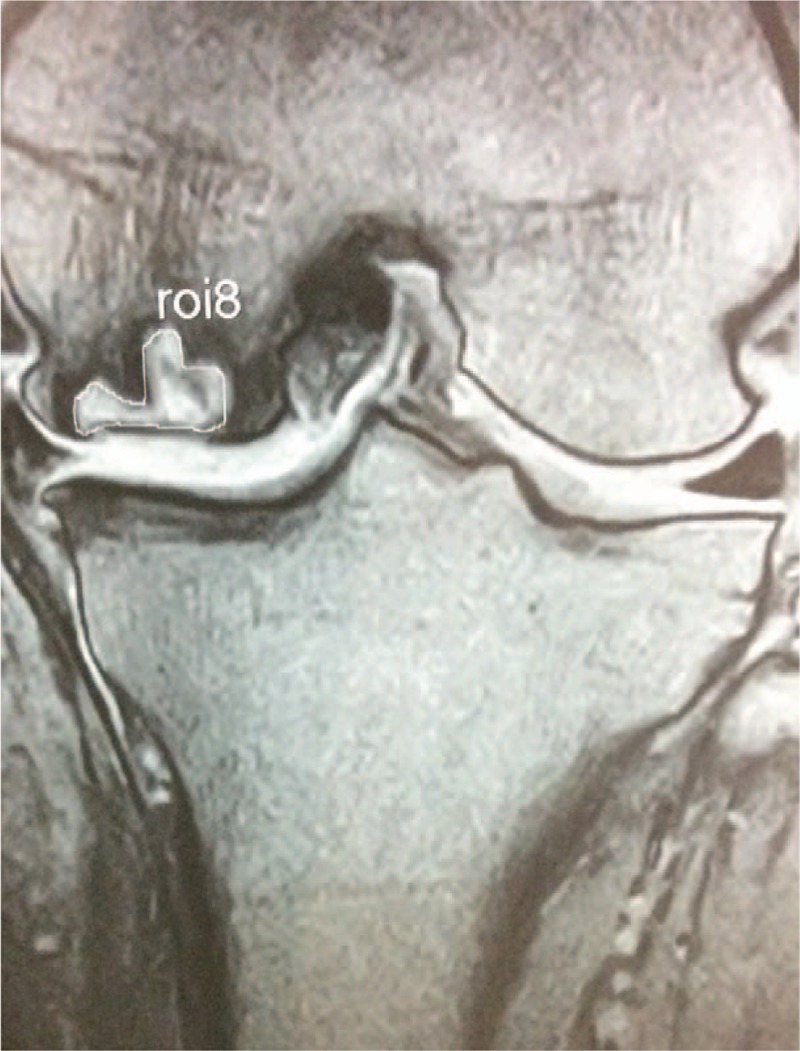
Six months after treatment with teriparatide. The size of the affected area has been decreasing gradually.

**Figure 4:**
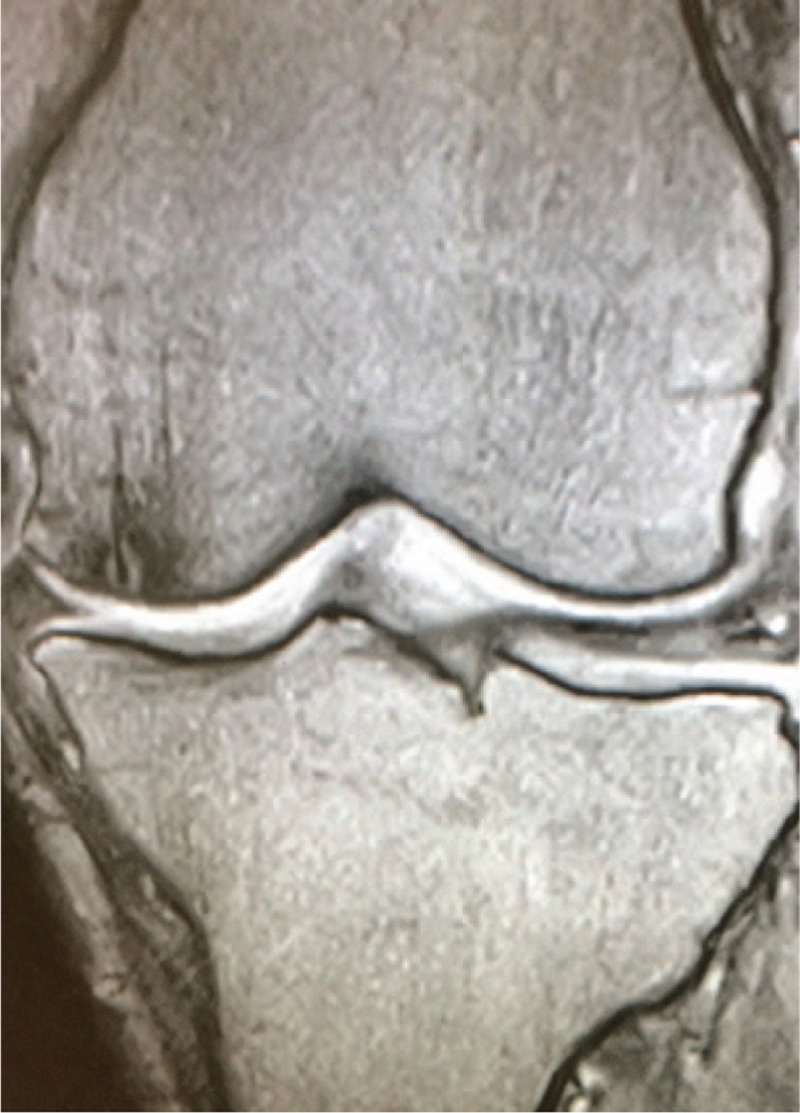
Twelve months after treatment with teriparatide. The size of the affected area has been decreasing dramatically.

**Figure 5:**
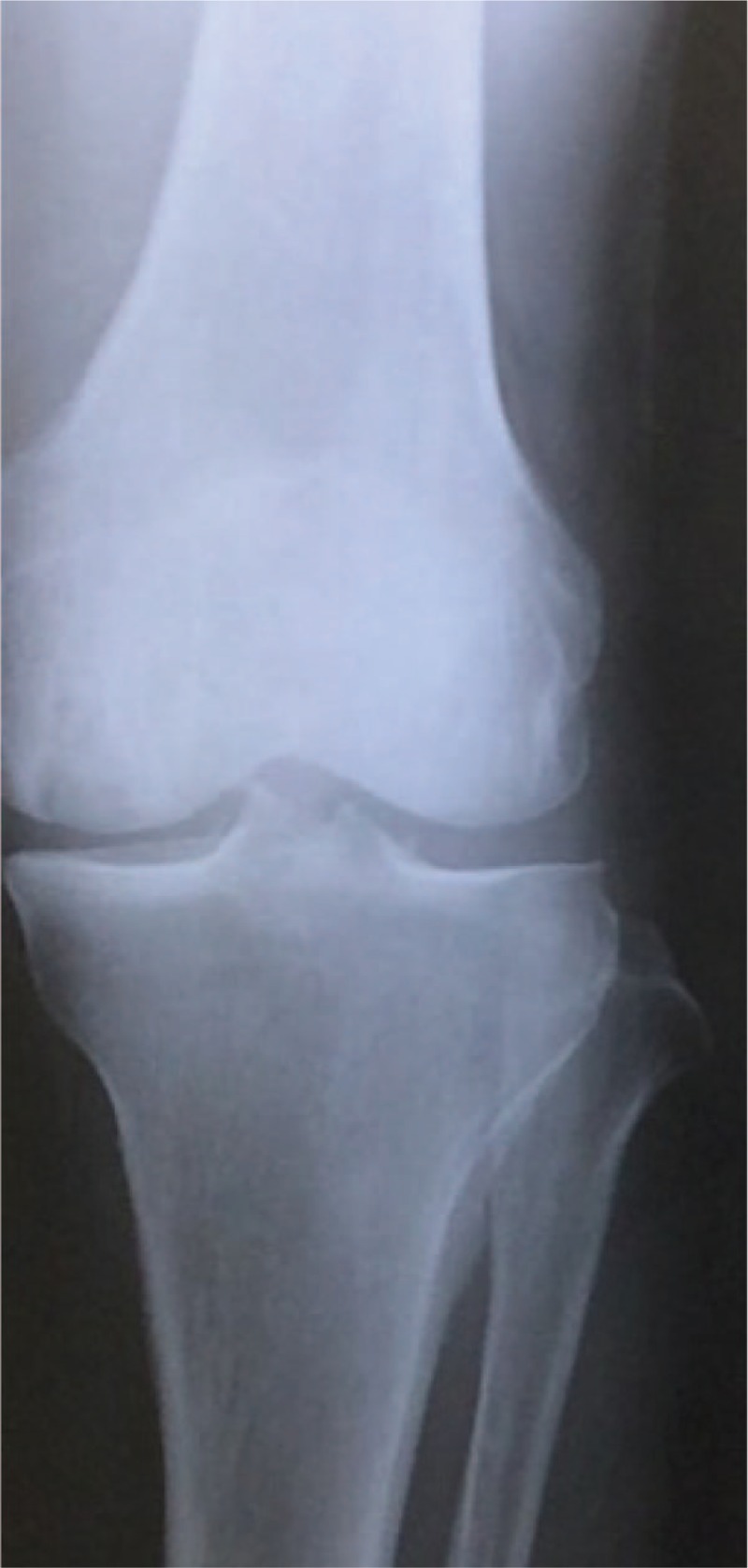
Six months after treatment with teriparatide. There is no collapse of subchondral bone accompanying the radiolucent zone in the femoral medial condyle.

**Figure 6:**
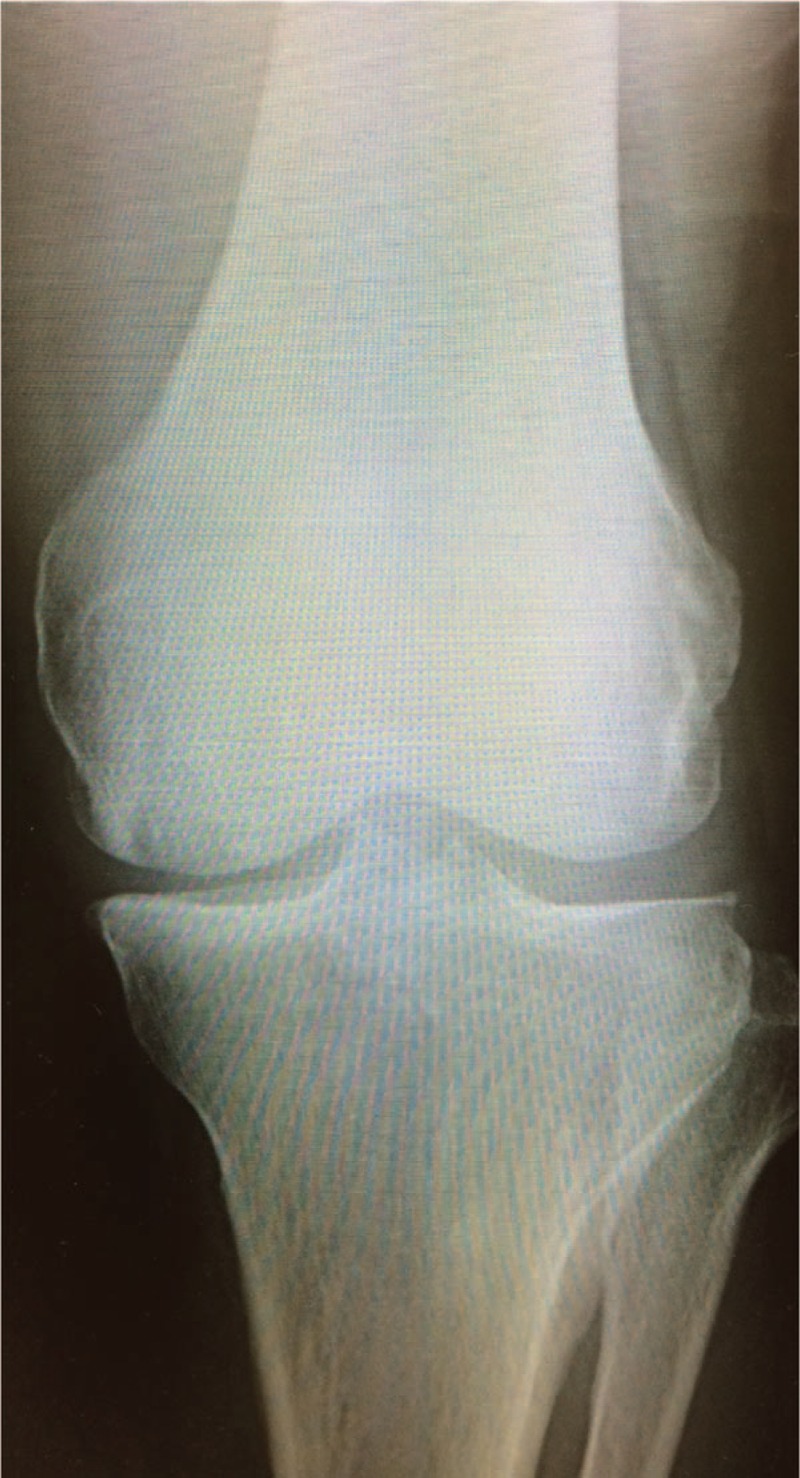
Twelve months after treatment with teriparatide. Although the size of the radiolucent zone in the femoral medial epicondyle has not changed, there is no sign of subchondral bone collapse.

A 74-year-old man complaining of left knee pain with no injury history visited our clinic for examination of his left knee joint and his osteoporotic status. Plain X-rays showed no osteoarthritis, which was classified as KL grade 1 (Fig. 1), whereas MRI suggested a SONK lesion with T1 low intensity and T2 high intensity (Fig. 2). He also had osteoporosis, with BMD under −2.5 standard deviation. Thus, daily teriparatide were initiated for his osteoporosis to regain knee function. After the start of daily teriparatide, his VAS score for pain decreased dramatically 1 month later, and MRI also showed that the size of the lesion decreased each 6 months and 12 months later (Figs. 3 and 4), although plain X-rays showed a radiolucent zone in the femoral medial epicondyle (Figs. 5 and 6).

### 3.2 Case 2

A 74-year-old woman who developed sudden onset pain involving both the left knee and the lumbar spine. Plain X-ray and dual DXA examinations showed mild osteoarthritis, KL grade 1, and osteoporosis. Her MRI suggested a SONK lesion, and laboratory findings such as bone turnover markers were in the normal range, which ruled out severe suppression of bone turnover or a high turnover status. Considering her medical history did not include treatment for osteoporosis, subcutaneous treatment with daily teriparatide was recommended. After she was started on daily teriparatide, her complaint reduced 1 month later and the size of the lesion on MRI decreased 6 months later.

### 3.3 Case 3

A 72-year-old woman who was diagnosed with osteoporosis complained of pain in her left knee. The FTA was almost 176° on plain X-ray, which also showed KL grade 1, implied pre-osteoarthritis stage. MRI showed a transient bone marrow edema lesion that looked like SONK. Considering her diagnosis of osteoporosis, daily teriparatide was recommended. After treatment with daily teriparatide, her complaint decreased dramatically, and the affected lesion on MRI also decreased as we previously mentioned same durations in case 1.

Baseline clinical characteristics of all 3 cases are shown in Table 1. Almost of all parameters were similar among of these patients. The sizes of the affected lesions changed on MRI in all 3 cases (Figs. 1–3). The areas of the low and high-intensity regions of the lesions on T2-weighted images decreased in size (Table 2), and the VAS also decreased from 7.8 to 2.3 over time (Table 3). The plain X-rays at the initial phase were classified as stage 1, and the average FTA was 175°. After 6 months of treatment, there was no evidence of progression, such as subchondral collapse and changes of the FTA (Table 3), although the affected areas showed radiolucent zones (Figs. 4–6). Bone turnover markers indicated similar teriparatide treatment patterns, an anabolic window with decreasing TRACP-5b levels, but a relatively slow decrease in P1NP levels (Table 4). Serum Ca was normal, with no hypercalcemia. With treatment, all patients had no restriction of weight bearing during their activities of daily living, and their complaints recovered gradually without any kinds of side effects.

**Table 1:**
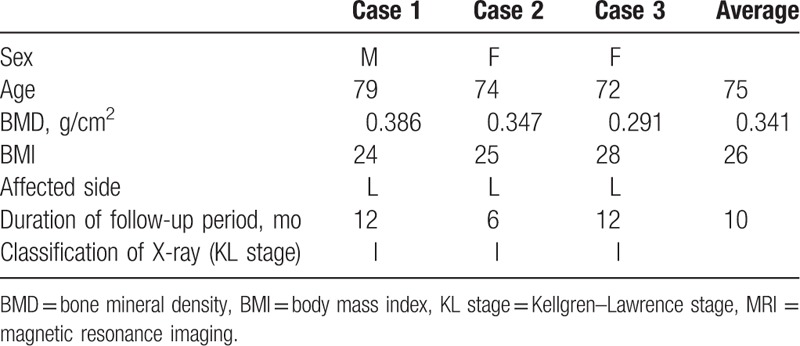
Baseline clinical characteristics of the 3 patients.

**Table 2:**
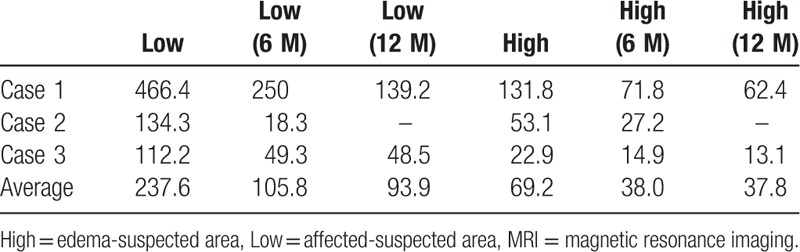
Chronological changes in the size of the affected area on T2-weighted MRI (mm^2^).

**Table 3:**
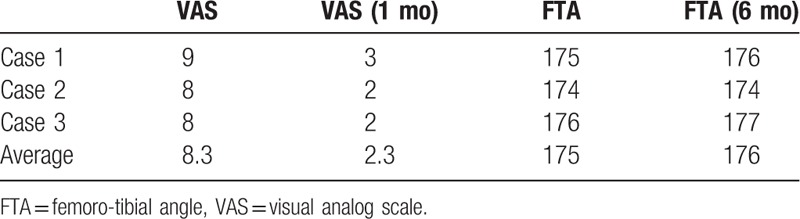
Chronological changes of VAS scores and the FTA.

**Table 4:**
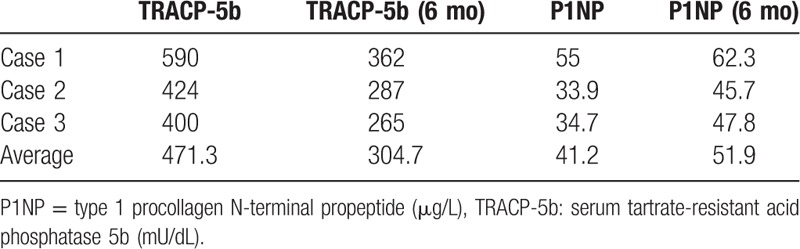
Changes of bone turnover markers over time.

## 4 Discussion

Ahlbäck advocated the concept of SONK in 1968,^[11]^ and its natural history began with sudden onset of progressive pain with tenderness over the medial femoral condyle and joint effusion without injury and significant radiographic finding in X-ray. Our 3 cases were defined as SONK by their symptom and appearances of plain X-ray examination according to Ahlbäck criteria. However, no clear consensus about the treatment of SONK has yet been established.^[12]^ The present cases showed good results with respect to the size of the affected lesion on MRI and the patients’ VAS scores over time. Geijer et al^[13]^ evaluated 1 case of SONK with repeated MRI over time, and he showed that the size of the affected lesion decreased gradually, although the affected lesion was still present for over 1 year. The present cases support this phenomenon, which may be attributed to the reconstruction of osteonecrosis of the femoral condyle. Moreover, he suggested the existence of the radiolucent zone of the affected femoral condyle on plain X-ray, which remained as a minimal subchondral lucency after 1 year and 3 months in the healing stage. The present cases also showed some radiolucent zones in the affected lesions. There is the possibility that the SONK lesions will be repaired in the near future according to this report.

There are some arguments about the treatment of SONK by bisphosphonates,^[8]^ which may prevent subchondral collapse of the femoral head. That mechanism was advocated because bisphosphonates have been shown to delay the resorption of revascularizing dead bone. In fact, this trial showed about 60% radiographic recovery of the medial femoral epicondyle with 70 mg of alendronate given once a week for a minimum of 6 months. However, some research has opposed treatment with bisphosphonates. This means that bisphosphonates might have positive and negative effects on this condition.^[14,15]^

To the best of our knowledge, there are no reports of the use of teriparatide for SONK in the English literature. There was a report that teriparatide promotes strong bone formation that can be used to promote the healing of nontraumatic osteonecrosis of the femoral head.^[16]^ Therefore, we focused on treatment with teriparatide in the SONK by administrating same dosage because these diseases were recognized as similar etiology. In the present cases, the complaints of knee pain of all 3 patients were dramatically reduced after 1 month of treatment based on their VAS scores, and the size of the affected areas on MRI decreased after 6 months. Considering these results and the changes of the normal bone turnover marker, teriparatide may be a useful treatment for SONK in the early stage and could prevent subchondral collapse.

Although there are some limitations because of the small number of patients and the lack of sufficient reports about the radiological findings in such cases, further investigation of more patients who have SONK is needed.

Finally, the effects of daily teriparatide in patients with SONK, which is an off-label use, were shown. All of them had no past history of treatment for osteoporosis, and they preferred subcutaneous treatment for osteoporosis rather than oral treatment because of poor adherence and compliance with oral medications. Daily teriparatide may be another option for treating this disease quickly without surgical procedures

In conclusion, conservative treatment of SONK with daily teriparatide resulted in marked improvement in patients’ complaints, and the size of the affected area decreased on MRI.

## Author contributions

**Conceptualization:** Akira Horikawa.

**Data curation:** Hiroyuki Kodama.

**Formal analysis:** Yuji Kasukawa.

**Project administration:** Michio Hongo.

**Supervision:** Naohisa Miyakoshi.

**Validation:** Yoichi Shimada, Akihisa Sano.

**Writing – original draft:** Akira Horikawa.
